# The moderating role of gender in the association between quality of social relationships and sleep

**DOI:** 10.1007/s10865-022-00286-6

**Published:** 2022-02-12

**Authors:** Zahra Mousavi, Mai-Lan Tran, Jessica L. Borelli, Amy L. Dent, Kate R. Kuhlman

**Affiliations:** 1 Department of Psychological Science, School of Social Ecology, University of California, 4201 Social and Behavioral Sciences Gateway, Irvine, CA 92697 USA; 2 Cousins Center for Psychoneuroimmunology, Semel Institute for Neuroscience & Human Behavior, University of California, Los Angeles, USA; 3 Institute for Interdisciplinary Salivary Bioscience, School of Social Ecology, University of California, Irvine, USA

**Keywords:** Sleep, Social support, Social strain, Health psychology, Objective sleep outcomes, Subjective sleep outcomes

## Abstract

To determine whether the association between perceived social support or strain in close relationships and sleep outcomes varies by gender. Participants were selected from the Biomarker projects of either the MIDUS II or MIDUS Refresher study if they were in a married-or married-like relationship and shared a bed with their partner (*N* = 989). A subsample also participated in a seven-day sleep study (*n* = 282). Perceived social support and strain from partner, family, and friends were examined by self-report questionnaires. We used the Pittsburgh Sleep Quality Index, sleep daily diary, and actigraphy to measure both subjective and objective sleep. Social support and strain were both associated with sleep outcomes. Specifically, higher social support was associated with fewer daily reports of light sleep and feeling more rested in the morning, while higher social strain was associated with higher clinical sleep disturbance. For women, but not men, social support was significantly associated with lower daily sleep disturbance while perceived social strain was significantly associated with higher daily sleep disturbance, lighter sleep, feeling less rested in the morning, lower sleep efficiency, and longer sleep onset latency. Mainly among women, social support and strain are associated with an important transdiagnostic health outcome–sleep–which may have implications for a wide range of health disparities. Interpersonal stressors may increase health risks differently for women compared to men and one mechanism that may link social relationships to long-term health outcomes is sleep.

**Supplementary Information:**

The online version contains supplementary material available at 10.1007/s10865-022-00286-6.

## Introduction

Social connection and quality of social relationships are important determinants of mental and physical health (Holt-Lunstad, 2018; Holt-Lunstad et al., 2010). Indeed, the mortality risk for having low social support is as large as risk factors such as obesity and physical inactivity (Holt-Lunstad et al., 2010), while social strain is related to chronic illness and higher mortality (Kiecolt-Glaser & Wilson, 2017; Vogli et al., 2007). Yet, our understanding of underlying processes through which social relationship quality, such as support and strain, impacts physical and mental health remains limited. Identifying such mechanisms would allow researchers to refine current treatment protocols and develop more effective interventions.

Social relationships may contribute to physical and mental health via sleep (e.g., Troxel et al., 2007). Sleep is a robust, transdiagnostic risk factor for a wide range of physical and mental health problems such as metabolic diseases, cardiovascular disease, cancer, post-traumatic stress disorder (PTSD), and ADHD (Harvey, 2008; Irwin, 2015) and contributes to all-cause mortality (Irwin, 2015). Sleep requires feeling physically and emotionally safe, which serves to down-regulate awareness and vigilance to the external world. Social relationships have an evolutionarily adaptive function of providing such a context (Dahl, 1996; Dahl & El-Sheikh, 2007; Troxel et al., 2009). Interpersonal security contributes to psychophysiological responses that could impact sleep onset and quality (Palagini et al., 2018). For instance, partner responsiveness, as a characteristic of interpersonal security, predicts lower arousal and consequently contributes to better sleep outcomes (Selcuk et al., 2017). Additionally, for most adults, sleep is a dyadic behavior. Seventy percent of American adults regularly sleep with a bed partner (National Sleep Foundation, 2013), making this a critical relationship context in which to explore social processes that affect sleep. Sleep behaviors are usually concordant among couples with parallel bed timing, wake timing, and the number of wakings (Meadows et al., 2009). Further, social interactions may impact sleep through their contribution to emotion or mood states that one experiences (Troxel et al., 2007). Therefore, understanding the contribution of social relationships to sleep may help researchers better understand how social relationships impact health outcomes in order to identify and isolate potential intervention targets to reduce the burden of physical and mental illness.

When examining sleep, it is important to assess its different facets by using both subjective and objective sleep outcomes. Subjective assessments of sleep are used to screen, diagnose, and monitor sleep complaints in clinical settings. Objective assessments of sleep, on the other hand, have the potential to assess constructs such as total sleep time, wake after sleep onset, and sleep efficiency which reflect underlying neurobiological processes that may be occurring outside of the individual’s awareness that suggest these two methods assess different dimensions of sleep and are additive rather than redundant (Aili et al., 2017; Buysse et al., 1991; Hsiao et al., 2018; Hughes et al., 2018; O’donnell et al., 2009; Zhang & Zhao, 2007). Further, subjectively- and objectively-measured sleep may predict different health outcomes. For instance, while observational and longitudinal epidemiologic studies suggest that poor objectively-measured sleep is a risk factor for diabetes, obesity, cancer (Luyster et al., 2012) and poorer retrospective and working memory (Cavuoto et al., 2016), subjectively-measured poor sleep quality is associated with a greater likelihood of death by suicide (Bernert et al., 2015) but not with risk of dementia (Lysen et al., 2018).

Compared to men, women are more likely to have disabling conditions such as arthritis, and depression (Crimmins et al., 2011). Women also report lower levels of self-rated health and more chronic health problems than men (Denton et al., 2004). Interpersonal stressors may increase health risks differently for women compared to men (Kiecolt-Glaser & Wilson, 2017). For instance, compared to men, women have more sensitive physiological responses (e.g. blood pressure, cortisol levels) to relationship interactions (Kiecolt-Glaser & Newton, 2001). Similarly, the association between social relationships and sleep may differ for men and women. Yet, with a few exceptions (El-Sheikh et al., 2015; Kane et al., 2014), the role of gender in the association between social relationships and sleep outcomes remains unexamined. Women report better sleep quality, and have higher sleep efficiency, and longer duration of sleep on days they have engaged in more self-disclosure to their partners (Kane et al., 2014). Additionally, the quality of interactions with their partner is associated with following-night sleep quality, sleep efficiency, and sleep onset latency among women, but not men (Hasler & Troxel, 2010). Although these findings suggest that women’s sleep may be more susceptible to social interactions with their partner compared to men, more research is needed to confirm this among other sources of social relationships (e.g., family and friends).

Perceived social support may contribute to more favorable sleep outcomes. Perceived social support is associated with lower clinical sleep disturbance (Chung, 2017; Kent et al., 2015; Liu et al., 2016; Stafford et al., 2017), better subjectively measured sleep outcomes (Ailshire & Burgard, 2012; Chung, 2017; Gosling et al., 2014), and better objectively measured sleep parameters (Chen et al., 2015; Troxel et al., 2010). However, the association between social support and sleep outcomes is not always consistent across literature, which may reflect the unique contribution of various sources of social support (i.e., partner vs. friends and family) to sleep outcomes. Specifically, the role of social relationships in sleep outcomes varies depending on whether partner as a source of social support and strain is included (Chen et al., 2015; Chung, 2017; El-Sheikh et al., 2015; Stafford et al., 2017). For example, a seven-day sleep study from the Midlife in the United States II (MIDUS II) study found that perceived social support from family (excluding spouse/partner) and friends predicted subjective, but not objective, sleep outcomes (Chung, 2017). In contrast, perceived support from one’s partner (Chen et al., 2015) and total social network, including partner, family, and friends (Troxel et al., 2010) is linked with actigraphy-measured sleep characteristics, but not with subjectively measured sleep outcomes such as daily sleep disturbances (Chen et al., 2015; Troxel et al., 2010). The inconsistencies linking social support and sleep outcomes may reflect the different potential sources of social support (i.e., partner vs. friends and family).

A smaller but growing literature shows that social strain may negatively contribute to sleep outcomes. Social strain such as relationship stress, social threats, and conflicts could contribute to emotional arousal, increase individuals' vigilance, interfere with sleep onset (Dahl, 1996), and negatively impact sleep outcomes (Ailshire & Burgard, 2012; Chen et al., 2015; El-Sheikh et al., 2013, 2015; Kent et al., 2015; Meadows & Arber, 2015; Rauer et al., 2010). Individuals with medium to high relationship distress with their partner experience poorer sleep compared to individuals with low distress (Meadows & Arber, 2015). Several previous studies examining the link between negative aspects of social relationships and sleep outcomes have focused on relationship aggression or intimate partner violence (El-Sheikh et al., 2013, 2015; Rauer et al., 2010). Higher aggression and violence in marital relationships predict more sleep disturbances (El-Sheikh et al., 2013, 2015; Rauer et al., 2010). Even mildly strained and demanding relationships could increase psychological distress (Durden et al., 2007) and consequently contribute to adverse sleep outcomes (Ailshire & Burgard, 2012; Chung, 2017; Gosling et al., 2014). Yet, very few studies have examined this link. Additionally, strain from other relationships such as family and friends may also increase one’s stress and anxiety (Hall et al., 2000), however, contribution of other sources of social strain to sleep outcomes remains unclear.

**Fig. 1:**
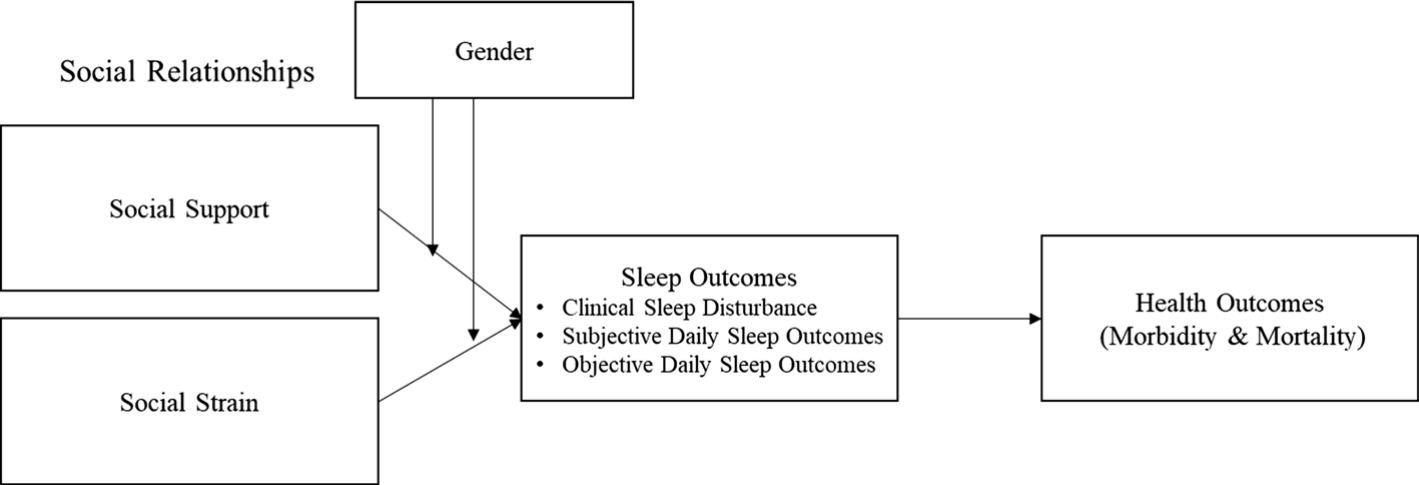
Theoretical model

The purpose of the present study was, therefore, to extend our understanding of the moderating role of gender in the association between quality of social relationships and sleep outcomes. To do this, we engaged the publicly available National Survey of Midlife Development in the United States (MIDUS). Previous publications using MIDUS Biomarker project have found poor sleepers (PSQI >  = 5, 41% of sample) to have lower average age, higher BMI, more chronic conditions, poorer self-rated health and lower socioeconomic status compared to normal sleepers (PSQI < 5) (Carroll et al., 2015). The role of social factors in sleep outcomes among older adults has also been explored. These studies show that social support such as partner responsiveness predicts better sleep outcomes (Selcuk et al., 2017) and social strain is associated with poorer sleep (Chung, 2017). Although sleep has been characterized in numerous publications in MIDUS datasets, the moderating role of gender in the association between quality of social relationships and sleep is lacking. Thus, we aimed to determine whether gender moderated the association between relationship support or strain from different people (spouse/partner, family, and friends) and sleep outcomes (Fig. 1). Specifically, in this study we aimed to determine (1) whether the association between social relationships and sleep outcomes varied by the relationship source of support or strain (spouse/partner, family, and friends), and (2) whether gender moderated those associations in MIDUS datasets (Fig. 1). We hypothesized that higher social support would be associated with lower PSQI score, indicating better global sleep, and better self-reported and actigraphy-measured daily sleep outcomes, and that higher social strain would be linked with worse global sleep, and daily subjective and objective sleep. Further, we hypothesized that the associations between support or strain and sleep would be stronger for women. In addition, the inconsistencies in the literature examining the link between quality of social relationships and sleep outcomes may be addressed by disaggregation of the role of each social relationship source in sleep outcomes (i.e., partner vs. family vs. friends). Therefore, exploratory post-hoc analyses sought to identify the unique contribution of different sources of support or strain to sleep outcomes.

## Methods

### Participants

**Fig. 2:**
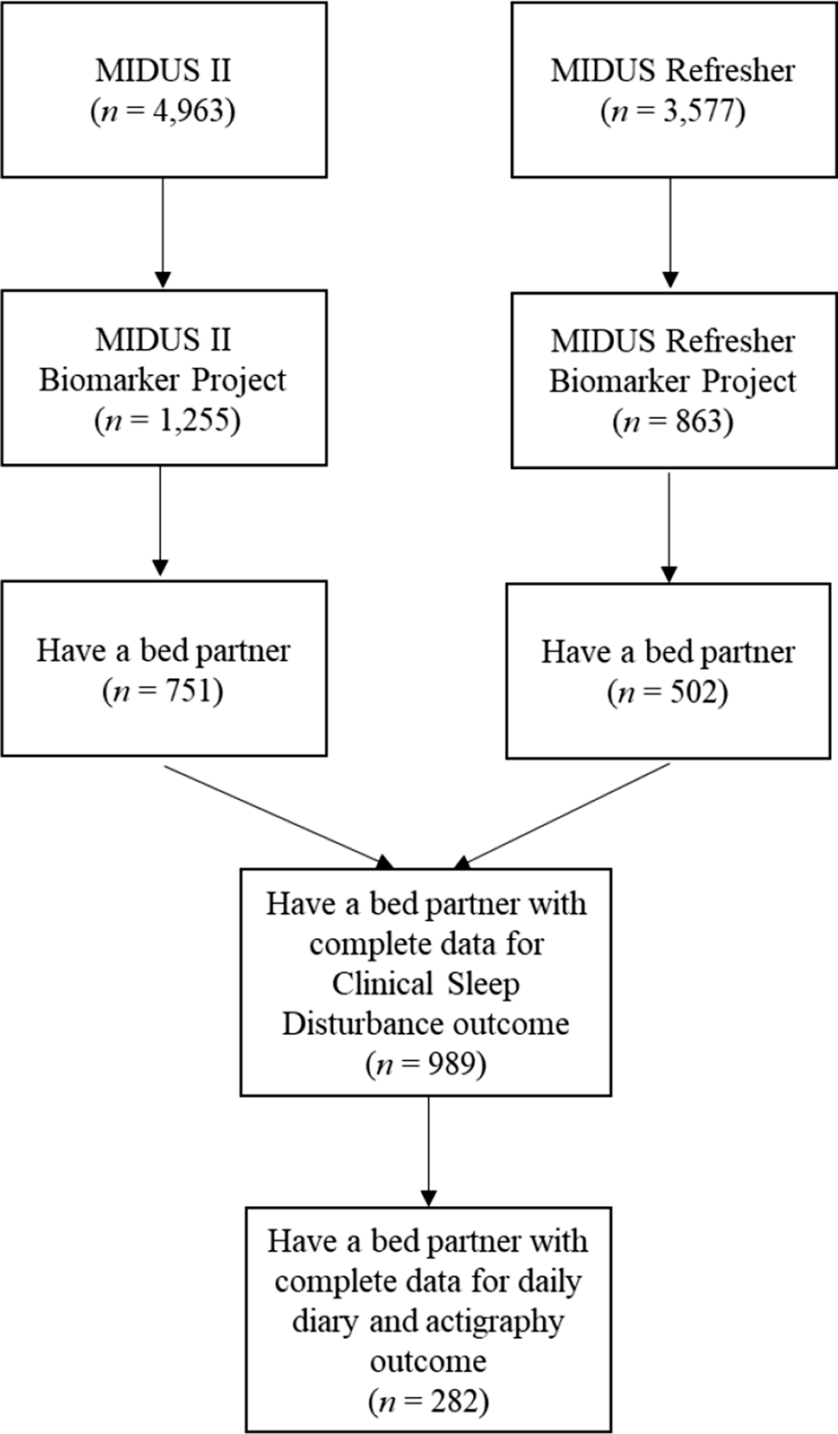
Flow of participants

The present study included participants from the MIDUS II Biomarker (*N* = 1,255) and MIDUS Refresher Biomarker (*N* = 863) studies. Imputation to mean was originally used by MIDUS team for daily diary and actigraphy variables that had missing values (Ryff et al., 2010). In the current study, we did not conduct further imputation for missing values. Specifically, when multiple variables have imputed values, the joint distribution and association may become complicated and increase bias (Horton & Kleinman, 2007). Only participants who met our inclusion criteria and had complete data for all primary variables and covariates were included in the analyses. The final analytical sample was 989 individuals for analyses predicting clinical sleep disturbance and 282 individuals for analyses predicting sleep measured via daily diary or actigraphy. Please see Fig. 2 for flow of participants in this study. Further, sleep is qualitatively different among people who share a bed with a partner vs. not (Drews et al., 2020). The vast majority (82.5%) of participants in this sample shared a bed with a partner. Thus, we restricted our analyses to individuals who shared a bed with their partner in order to reduce the risk of type 1 errors.

**Fig. 3:**
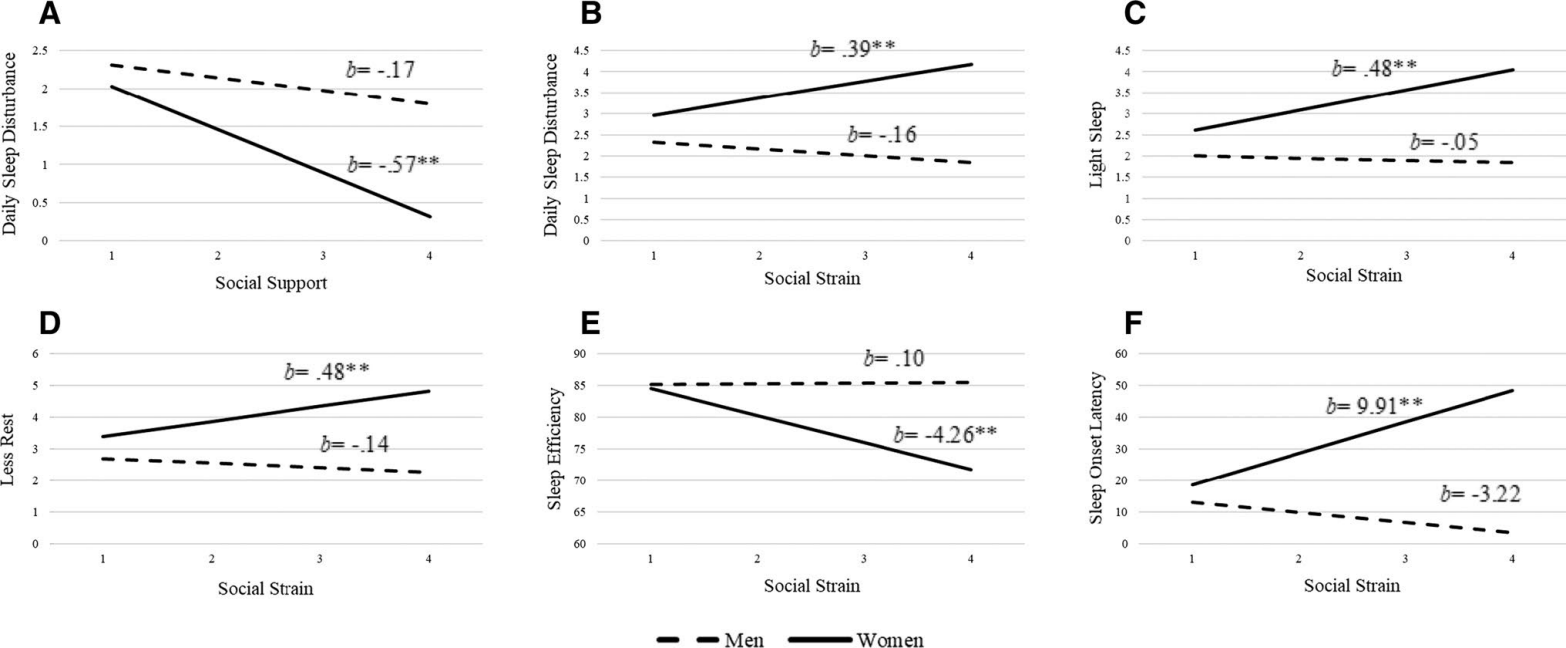
Moderating role of gender in the relationship between social support and strain and daily sleep outcomes

The data for this study came from the MacArthur study on Midlife Development in the United States (MIDUS). The original MIDUS I study (1995–1996) is a national probability sample of noninstitutionalized, English-speaking adults in the contiguous United States obtained by random-digit telephone dialing. Of the 7,108 participants in MIDUS I, 4,963 individuals participated in wave II. The MIDUS II Biomarker project (2004–2009) was conducted 5 to 64 months after wave II. A subsample of these respondents participated in a seven-day daily diary and actigraphy sleep study. The MIDUS Refresher study (2011–2014; El-Sheikh et al., 2013) was conducted using a novel sample with the same methods as MIDUS II. Further details regarding the sample and methods of the study have been reported in prior reports of the study (Ryff, 2017; Weinstein, 2017).

### Procedure

Participants in the MIDUS II Biomarker and MIDUS Refresher Biomarker projects completed a self-report questionnaire for demographics and psychosocial assessments, including the Pittsburgh Sleep Quality Index (PSQI) (Buysse et al., 1989) in their two-day visit to the laboratory. After completion of the Biomarker project, participants were invited to participate in a seven-day sleep study. After completing informed consent to participate in the sleep study, participants were sent home with instructions and study materials. The instructions included completing a daily sleep diary and wearing a wrist actigraph for seven consecutive days.

### Measures

#### Social support and strain

**Social support **Perceived social support was measured using four items that participants answered for each of the following relationship categories a) spouse/partner, b) family members (excluding spouse/partner), and c) friends. An example of these items was: “How much do your (spouse/partner; family; and friends) really care about you?” Social support from spouse/partner included two additional items: (i) How much does he or she appreciate you? (ii) How much can you relax and be yourself around him or her? Participants answered all the items on a 4-point scale ranging from 1 to 4 (support items: 1 = “a lot”; 4 = “not at all”). All the items were then reverse coded so that higher scores indicated higher support. To compute total social support, the partner/spouse, family, and friends scores were averaged. Internal reliability for each social support variable was high for the total sample and the sub-sample included in daily diary and actigraphy analyses, respectively: spouse/partner support (0.89 and 0.88), family support (0.84 and 0.86), friend support (0.87 and 0.88).

**Social strain** Perceived social strain was measured via participant self-report on four items for each of the following relationship categories a) spouse/partner, b) family members, and c) friends. An example of these items was: “How often do your (spouse/partner, family, or friends) make too many demands on you?” Participants answered all items on a 4-point scale ranging from 1 to 4: 1 = “often”, 4 = “never”). All items were then reverse coded so that higher scores indicated higher strain. To compute total social strain data, partner/spouse, family, and friends scores were averaged. Internal reliability for each strain variable was acceptable for the total sample and the sub-sample included in daily diary and actigraphy analyses, respectively: spouse/partner strain (0.87 and 0.85), family strain (0.77 and 0.76), and friend strain (0.79 and 0.80).

#### Sleep outcomes

**Clinical sleep disturbance** Participants completed the Pittsburgh Sleep Quality Index (PSQI) (Buysse et al., 1989) on day one of the laboratory visits as part of a large questionnaire battery. This self-report instrument is a 19-item scale that assesses sleep quality over the past month. This instrument yields a global score on sleep disturbance with a possible range of 0–21 with lower scores representing better sleep (Buysse et al., 1989). A global sleep score of five or greater indicates a likely clinical sleep disturbance (Buysse et al., 1989). This instrument is well-validated and reliable with 98.7% sensitivity and 84.4% specificity distinguishing insomnia patients versus controls (Buysse et al., 1989).

**Subjective daily sleep** Subjective daily sleep was measured using a seven-day sleep daily diary, which included a morning and evening section. Participants were instructed to complete the morning questionnaire upon awakening, waiting no more than 10 min and the evening questionnaire before going to sleep. An average score was computed for each daily sleep outcome across the seven days.

**Daily sleep disturbances** Participants reported their overall quality of sleep every morning on a 5-point Likert scale (1 = “very good”, 5 = “very poor”) by rating: “Overall quality of sleep last night.”

**Light sleep** Participants rated how deeply they slept the previous night every morning on a 5-point Likert scale (1 = “very deeply”, 5 = “very lightly”) by answering, “How deeply you slept last night?”.

**Feeling less-rested** Participants rated how well-rested they felt every morning on a 5-point Likert scale (1 = “well-rested”, 5 = “poorly rested”) by answering, “How well-rested you feel this morning?”.

**Objective daily sleep** Each participant wore a sensor on the wrist of the non-dominant arm that allowed tracking movement (i.e., actigraphs) for seven days. The actigraph used in MIDUS was the MiniMitter Actiwatch 64. Actiwatches were programmed to begin collecting data at 7:00 am on the start day until the end of the study. The Actiware software (Versions 5 or 6) was used to detect sleep based on 30-s epochs in order to generate summary statistics about the participants’ sleep. A more detailed explanation of the procedure of coding activity data can be found elsewhere (Ryff, 2017; Weinstein, 2017). Sleep efficiency, sleep onset latency, and total sleep time were computed by the Actiware program algorithms based on the rest intervals. Data for the sleep indices were averaged across the seven days of data collection.

#### Covariates

Based on the previous literature, several variables have been linked to sleep outcomes such as major health events (Shankar et al., 2010), age (Ohayon et al., 2004), and working status (Lallukka et al., 2010). These variables were assessed and included as covariates in the regression model for predicting clinical sleep disturbance. In addition, the number of caffeinated drinks (Clark & Landolt, 2017), the number of alcoholic drinks (Ebrahim et al., 2013), minutes of moderate or vigorous exercise (Driver & Taylor, 2000), and minutes of napping (Dhand & Sohal, 2006) during the day were also averaged across the 7 days of data collection and included as covariates for daily diary and actigraphy sleep outcomes.

### Data analysis

All predictor and outcome variables were examined for normality and heteroscedasticity. Sleep onset latency and sleep efficiency were winsorized to three standard deviations (3SD) from the mean. 8 values were winsorized for sleep onset latency and 9 values were winsorized for sleep efficiency. To determine the main effect of total social support and social strain on clinical, daily subjective, and objective sleep outcomes, we conducted multiple regression models predicting sleep outcomes from total social support and strain while accounting for the covariates. If the association between perceived social support and strain with any of the sleep outcome variables was significant, we then examined the unique contribution of each potential source of social support and strain (i.e., spouse/partner, family, friends) as predictors of variance in each sleep outcome.

We then examined whether gender moderated the association between total social support and sleep outcomes, then total social strain and sleep outcomes. All predictors were mean-centered before the analysis. In models where the estimated interaction between support/strain and gender was reliable at *p* < 0.05, we estimated the association between social support or strain with sleep outcomes separately for men and women using PROCESS (Version 3.4) in SPSS (Hayes, 2012).

## Results

More than half (54.4%) of our sample reported clinically meaningful sleep disturbance. There were significant bivariate associations between perceived social support and strain with clinical sleep disturbance (all *p*s < 0.04), daily sleep disturbances, light sleep, feeling less-rested, and lack of alertness measured with daily diaries (all *p*s < 0.03).

### Social support and strain as predictors of clinical sleep disturbance

The model predicting clinical sleep disturbance from total social support and strain while controlling for the major health events, age, and employment status accounted for 3% of the variance in clinical sleep disturbance as measured by PSQI, *Adj. R*^*2*^ = 0.03, *F*(5,983) = 6.40,* p* < 0.001. Greater perceived social strain was associated with higher clinical sleep disturbance, *b* = 0.78, *SE* = 0.26, *p* = 0.003. Among all the sources of perceived social strain, only strain from family was significantly associated with a higher global sleep score, *b* = 0.71, *SE* = 0.21, *p* = 0.001. Gender did not moderate the association between social support or strain and clinical sleep disturbance (Table 1).

**Table 1:** Adjusted estimates predicting clinical sleep disturbance from perceived social support and strain, gender, and their interactions

	Clinical sleep disturbance			
	*b(SE)*	*P*	95% CI	
			*LL*	*UL*
*Model 1*^*a*^				
Total perceived support	−.57 (.31)	.07	−1.20	.06
Gender	.91 (.21)	< .001	.50	1.31
Total perceived support * Gender	−.24 (.48)	.62	−1.19	.71
*R*^2^	.05			
F	7.41	< .001		
*Model 2*^*b*^				
Total perceived strain	.60 (.35)	.09	−.09	1.30
Gender	.89 (.20)	< .001	.49	1.29
Total perceived strain * Gender	.14 (.47)	.77	−.79	1.06
*R*^2^	.05			
F	7.38	< .001		

*n* = 989; ^a^Adjusted for social strain, major health events, age, employment status

^b^Adjusted for social support, major health events, age, employment status

### Social support and strain as predictors of subjective daily sleep outcomes

The multiple regression models predicting daily sleep disturbances, light sleep, and feeling less−rested from total social support and strain, while controlling for all the covariates, accounted for 7% of the variance in daily sleep disturbances, *Adj. R*^*2*^ = 0.07, *F*(9,272) = 3.51,* p* < 0.001, 4% of variance in sleep depth, *Adj. R*^*2*^ = 0.04, *F*(9,272) = 2.19,* p* = 0.023, and 10% of variance in feeling less−rested*, Adj. R*^*2*^ = 0.10, *F*(9,272) = 4.54,* p* < 0.001*.* While only greater perceived social support was significantly associated with lower daily sleep disturbances, *b* = -0.30, *SE* = 0.10, *p* = 0.004, and feeling more rested, *b* = -0.29, *SE* = *0.1*0, *p* = 0.004, both perceived social support and social strain were associated with light sleep, *b* = -0.24, *SE* = *0.1*0, *p* = 0.022, and, *b* = 0.26, *SE* = 0.11, *p* = 0.023, respectively. The association between social support and strain, and subjective sleep outcomes were not uniquely driven by any relationship type (spouse/partner, family, and friends).

Gender moderated the link between perceived social support and daily sleep disturbances, *b* = -0.40, *SE* = 0.19, *p* = 0.04 (Table 2). Women, *b* = -0.57, *SE* = 0.16, *p* < 0.001, but not men, *b* = -0.17, *SE* = 0.13, *p* = 0.20, who reported more social support had less daily sleep disturbances. Gender also moderated the link between perceived social strain and daily sleep disturbances, *b* = 0.56, *SE* = 0.21, *p* = 0.007 (Table 2). Specifically, higher social strain was associated with higher sleep disturbances for women, *b* = 0.39, *SE* = 0.15, *p* = 0.008, but not men, *b* = -0.16, *SE* = 0.16, *p* = 0.31. Please see Fig. 3.

**Table 2:** Adjusted estimates predicting subjective daily sleep outcomes from perceived social support and strain, gender, and their interactions

	Daily sleep disturbance				Light sleep				Less rested			
	*b(SE)*	*p*	95% CI		*b(SE)*	*p*	95% CI		*b(SE)*	*p*	95% CI	
			*LL*	*UL*			*LL*	*UL*			*LL*	*UL*
*Model 1*^*a*^												
Total perceived support	−.17 (.13)	.20	−.43	.09	−12 (.13)	.33	−.38	.13	−.17 (.13)	.17	−.42	.07
Gender	.12 (.09)	.20	−.06	.30	.11 (.09)	.23	−.07	.29	.10 (.09)	.25	−.07	.28
Total perceived support * Gender	−.40 (.19)	.04	−.78	−.02	−.35 (.19)	.07	−.73	.03	−.36 (.19)	.06	−.73	.01
*R*^2^	.12				.08				.14			
F	3.38	< .001			2.19	.015			4.14	< .001		
*Model 2*^*b*^												
Total perceived strain	−.16 (.16)	.31	−.48	.15	−.05 (.16)	.77	−.35	.26	−.14 (.15)	.35	−.44	.16
Gender	.09 (.09)	.34	−.09	.26	.08 (.09)	.36	−.09	.25	.07 (.09)	.41	−.10	.24
Total perceived strain * Gender	.56 (.21)	.007	.15	.97	.53 (.20)	.01	.13	.92	.62 (.20)	.002	.23	1.01
*R*^2^	.13				.09				.16			
F	3.68	< .001			2.52	.005			4.79	< .001		

*n* = 282; ^a^Adjusted for social strain, major health events, age, employment status, number of caffeinated drinks, number of alcoholic drinks, minutes of moderate or vigorous exercise, and length of nap time (minutes)

^b^Adjusted for social support, major health events, age, employment status, number of caffeinated drinks, number of alcoholic drinks, minutes of moderate or vigorous exercise, and length of nap time (minutes)

Gender moderated the association between perceived social strain and light sleep, *b* = 0.52, *SE* = 0.20, *p* = 0.01 (Table 2). Women, but not men with more perceived social strain reported lighter sleep, *b* = 0.48, *SE* = 0.14, *p* = 0.001, for women and, *b* = -0.05, *SE* = 0.16, *p* = 0.77 for men. Finally, gender moderated the link between perceived social strain and feeling less-rested, *b* = 0.62, *SE* = 0.20, *p* = 0.002 (Table 2). Women, but not men, with more perceived social strain reported a higher average of feeling less-rested in the morning, *b* = 0.48, *SE* = 0.14, *p* = 0.001 for women and *b* = -0.14, *SE* = 0.15, *p* = 0.35 for men. Gender did not moderate the link between perceived social support and light sleep, *b* = -0.35, *SE* = 0.19, *p* = 0.07, or feeling less-rested, *b* = -0.36, *SE* = 0.19, *p* = 0.056 (Table 2). Please see Fig. 3.

### Social support and strain as predictors of objective daily sleep outcomes

Total perceived support and strain did not account for a significant amount of variance in sleep efficiency, sleep onset latency, and sleep time. However, gender moderated the association between social strain with objective daily sleep outcomes (Table 3). Specifically, gender moderated the link between perceived social strain and sleep efficiency, *b* = -4.36, *SE* = 2.19, *p* = 0.048; higher social strain was associated with lower sleep efficiency for women, *b* = -4.26, *SE* = 1.57, *p* = 0.007, but not men, *b* = 0.10, *SE* = 1.70, *p* = 0.95.^1^ Gender also moderated the link between perceived social strain and sleep onset latency, *b* = 13.13, *SE* = 5.84, *p* = 0.025. Women, but not men, with more perceived social strain had higher sleep onset latency, *b* = 9.91, *SE* = 4.18, *p* = 0.018 for women and *b* = -3.22, *SE* = 4.52, *p* = 0.48 for men. Please see Fig. 3.^2^

**Table 3:** Adjusted estimates predicting objective daily sleep outcomes from perceived social support and strain, gender, and their interactions

	Sleep efficiency				Sleep onset latency				Sleep time			
	*b(SE)*	*p*	95% CI		*b(SE)*	*p*	95% CI		*b(SE)*	*p*	95% CI	
			*LL*	*UL*			*LL*	*UL*			*LL*	*UL*
*Model 1*^*a*^												
Total perceived support	−4.08 (1.39)	.0035	−6.81	−1.35	7.94 (3.71)	.03	.64	15.24	−22.09 (11.21)	.05	−44.17	−.02
Gender	3.44 (.97)	< .001	1.52	5.36	−6.87 (2.60)	.01	−11.99	−1.74	40.41 (7.88)	< .001	24.89	55.92
Total perceived support * Gender	3.92 (2.06)	.058	−.14	7.98	−8.83 (5.52)	.11	−19.70	2.03	11.64 (16.70)	.49	−21.23	44.51
*R*^2^	.15				.09				.19			
F	4.51	< .001			2.52	.005			5.70	< .001		
*Model 2*^*b*^												
Total perceived strain	.10 (1.70)	.95	−3.24	3.44	−3.22 (4.52)	.48	−12.12	5.68	13.09 (13.68)	.34	−13.85	40.03
Gender	3.75 (.96)	< .001	1.86	5.65	−7.59 (2.56)	.003	−12.63	−2.55	41.42 (7.75)	< .001	26.16	56.67
Total perceived strain * Gender	−4.36 (2.19)	.048	−8.68	−.04	13.13 (5.84)	.02	1.63	24.63	−29.63 (17.67)	.09	−64.42	5.17
*R*^2^	.16				.10				.19			
F	4.55	< .001			2.77	.002			5.96	< .001		

*n* = 282; ^a^Adjusted for social strain, major health events, age, employment status, number of caffeinated drinks, number of alcoholic drinks, minutes of moderate or vigorous exercise, and length of nap time (minutes)

^b^Adjusted for social support, major health events, age, employment status, number of caffeinated drinks, number of alcoholic drinks, minutes of moderate or vigorous exercise, and length of nap time (minutes)

## Discussion

In this study, we characterized the association between perceived social support and strain from different sources (partner, family, and friends) with both subjective and objective sleep outcomes and investigated whether gender moderated this association. Overall, both perceived social support and strain predicted subjectively measured sleep outcomes. Specifically, higher social support was associated with lower daily sleep disturbances, fewer daily reports of light sleep, and feeling more rested in the morning, while higher social strain was associated with higher clinical sleep disturbance, and more daily reports of light sleep. The associations between perceived social support with daily sleep disturbances and social strain with light sleep were only significant for women. Additionally, women with higher perceived social strain reported higher sleep disturbances, feeling less rested in the morning, lower sleep efficiency, and longer sleep onset latency. These findings have important implications for both sleep and relationship research, as well as interventions focused on social relationships.

Social support predicts day-to-day subjective sleep outcomes. Consistent with previous studies (Pow et al., 2017) our findings suggest that higher social support predicts better subjective daily sleep outcomes. It is well-established that social support is related to better health and later mortality (Holt-Lunstad et al., 2010). Considering the importance of sleep as a transdiagnostic process that influences physical and mental health (Harvey, 2008; Irwin, 2015), it is important to identify modifiable determinants of sleep such as quality of social relationships to reduce the burden of physical and mental illness. The difference in findings between clinical sleep disturbance and daily subjective reports of sleep may indicate that daily diary is a more sensitive measure of the role of interpersonal factors in sleep. Clinical sleep disturbance, measured by PSQI, is used in medical settings as a screening measure to identify people with insomnia and other sleep disorders (Buysse et al., 1989), and the daily diary approach provides more nuanced characterizations of the antecedents and consequences of sleep outcomes (Kalmbach et al., 2017; Pillai et al., 2014). Improving social support may be particularly important to prevent daily sleep disturbances in order to interrupt the pathogenesis of illness states such as insomnia. Future research may benefit from experimentally studying the contribution of social support to sleep outcomes, as a transdiagnostic outcome impacting health, in order to identify the modifiable determinants of sleep.

We also observed a positive association between social strain and clinical sleep disturbance. Gender did not moderate this association. However, the association between perceived social strain with daily subjective and objective sleep outcomes varied as a function of gender. Specifically, only among women, perceived social strain was associated with daily sleep disturbances, daily reports of light sleep, and feeling less-rested, lower sleep efficiency, and longer sleep onset latency. Subjective and objective sleep outcomes are important risk factors for insomnia, depressive and anxiety symptoms (Kalmbach et al., 2017) and prevention, early detection and treatment of sleep disturbances may interrupt this cycle at early stages. Overall, compared to men, women report more subjective sleep problems (Friedman, 2011; van den Berg et al., 2009) and are at greater risk for various mental health problems such as depression (Kessler et al., 2005). This gender difference is consistent with previous research showing that gender-related differences such as interpersonal stressors may increase mental and physical health risks for women compared to men (Hasler & Troxel, 2010; Kiecolt-Glaser & Wilson, 2017). Based on our findings, it is plausible that women may benefit more from interventions targeting social strain and conflict to improve subjectively measured sleep outcomes. The association found between perceived social strain and day-to-day subjective and objective sleep outcomes among women may reflect arousal (e.g., post-conflict rumination) in response to perceived strain (e.g., Driver & Taylor, 2000; Ebrahim et al., 2013). The association between strain and sleep may indicate a deficiency in the recovery of nervous and biological systems that regulate stress response (Kalmbach et al., 2017). In other words, social strain and conflict may contribute to rumination as a hyperactivating emotion regulation strategy (Mikulincer et al., 2003), amplify arousal in nervous and biological systems, and delay their recovery from stress, which in turn impacts sleep outcomes. Future research may benefit from investigating the moderating role of gender in the impact of social strain on magnitude and duration of physiological arousal, particularly in the context of sleep-onset latency.

Another potential avenue for future research is the role of menopause as a plausible biological mechanism linking social strain and sleep among women. In the current study, 248 of the female participants from females in the total sample (n = 473) provided information about their menopause status (60.5% post-menopausal). Previous studies have shown a significant increase in sleep disruption among perimenopausal participants compared to the premenopausal group (Baker et al., 1997). Further, perimenopausal subjects experience significantly less sleep due to longer and more arousals. Therefore, future research may benefit from examining menopause as a biological mechanism explaining the difference between men and women in the association between social strain and daily sleep outcomes.

Interestingly, gender did not significantly moderate the association between social strain and clinical sleep disturbance. In other words, men appear to be also vulnerable to social strain when looking at clinical sleep outcomes. This finding may indicate that although there was no association between strain and day-to-day sleep outcomes among men, in the long-term, chronic social strain such as relationship stress may still contribute to adverse sleep outcomes in men as well.

When we probed the unique contributions of support and strain from partner, family, and friends to sleep outcomes, only strain from family contributed to the higher clinical sleep disturbance. MIDUS datasets gave us a unique opportunity to disentangle the contribution of family relationships vs. spouse/partner and friends to sleep outcomes. This may reflect the unique contribution of various social relationships to different health outcomes. Our findings are in line with previous research showing stronger association between family relationships and health compared to peer relationships (Shor et al., 2013). Demanding family relationships may add to the problems and stress that one experiences, and lead to greater stress and anxiety (Hall et al., 2000). Differentiating among close relationships in ways that better capture the complex social network in which health processes unfold across the lifespan will be critical to developing interventions that enhance health through social integration.

### Limitations

This study should be interpreted in the context of its limitations. First, this study is limited by its cross-sectional design. Whether social support and strain are causally linked to sleep outcomes remains unknown; it is plausible that the association between social relationships and sleep is bidirectional. Sleep parameters such as disrupted sleep and daytime fatigue could also contribute to lower relationship quality (Ben Simon et al., 2020; Brooks Holliday & Troxel, 2017). It is important to note that the interaction between strain and gender was non-significant for sleep efficiency and sleep onset latency after removing a small number of influential participants from the analysis. This may suggest that there are additional factors to consider in these processes. Several studies have documented robust differences in sleep across ethnic and racial groups. For example, African Americans compared to Caucasian Americans have poorer sleep continuity and duration (Yip et al., 2020). We were underpowered to detect whether our observations differed by participant race/ethnicity due to the over-representation of non-Hispanic white participants (91%). Exploration within more ethnically diverse samples is needed. Whether social support and strain can explain these racial and ethnic differences remain unknown. Future studies could also adopt multilevel modeling to focus on more nuanced within-person fluctuations in sleep and quality of social relationships over time or context, while also probing differences based on demographic characteristics. Finally, the average age of our sample was 52.95 years (SD = 12.13). Considering specific, and adaptive roles of social relationships in different lifespan contexts (Mikulincer et al., 2003) and changes in sleep quality and duration across the lifespan (Buysse et al., 1991) we do not expect the association between social relationships and sleep outcomes to be constant across the lifespan.

## Conclusion

Quality of social relationships may be a modifiable factor to target for the benefit of sleep and overall health among women. Social relationships are associated with an important transdiagnostic outcome, sleep, which may have implications for a wide range of health disparities. Improved sleep outcomes may enhance daily functioning, physical health (During & Kawai, 2017; Irwin, 2015), and mental health outcomes (Harvey, 2008). In light of our results, mainly among women, the quality of social relationships is associated with sleep outcomes. Social relationships may impact health risks differently for women compared to men and one mechanism that may link social relationships to long-term health outcomes is sleep. If supported with future experimental studies, these findings may have important implications for identifying potential intervention targets for the improvement of mental and physical health.

## Supplementary Information

Below is the link to the electronic supplementary material.

Supplementary file1 (PDF 123 KB)

## Footnotes

1 After excluding participants with influential values (|DFFITS|> 2, n = 4), gender no longer moderated the association between social strain and sleep efficiency (*b* = -3.34, *SE* = 2.33, *p* = .15).

2 After excluding participants with influential values (|DFFITS|> 2, n = 14), gender no longer moderated the association between social strain and sleep onset latency (*b* = 5.61, *SE* = 5.63, *p* = .32).
