# Supplementary material for: The moderating role of gender in the association between quality of social relationships and sleep
Source: J Behav Med. 2022 Feb 12;45(3):378–90. doi: 10.1007/s10865-022-00286-6 (PMC9160110; doi:10.1007/s10865-022-00286-6)
Supplement: Supplementary file 1 — Supplementary file1 (PDF 123 KB) [file 10865_2022_286_MOESM1_ESM.pdf]

Table 1
Sociodemographic Characteristics of Participants

	Global sleep score			Global sleep score and share bed			Daily diary and actigraphy			Daily diary and actigraphy and share bed		
	M(SD)	*n*	*%*	*M(SD)*	*n*	*%*	*M(SD)*	*n*	*%*	*M(SD)*	*n*	*%*
Gender												
Male		624	52		516	52.2		168	50		141	50
Female		575	48		473	47.8		168	50		141	50
Marital status												
Married		1110	92.6		945	95.6		318	94.6		272	96.5
Separated		13	1.1		2	.2		1	.3		1	.4
Divorced		43	3.6		20	2.0		10	3.0		4	1.4
Widowed		4	.3		2	.3		0	0		0	0
Never married		29	2.4		19	1.9		7	2.1		5	1.8
Age	53.84 (12.28)			52.95 (12.13)			52.46 (12.19)			51.25 (11.98)		
Share bed with partner[a]		989	82.5		989	100		282	83.9		282	100
Highest level of education												
< high school		29	2.4		20	2		13	3.9		11	3.9
GED or graduated from high school		208	17.3		168	17		66	19.6		53	18.8
Some college		326	27.2		262	26.5		100	29.8		81	28.7
Bachelor’s degree		308	25.7		253	25.6		84	25		73	25.9

Some graduate school or graduate degree		326	27.2		284	28.7		73	21.7		64	22.7
Employment status												
Currently employed		659	55		558	56.4		184	54.8		165	58.5
Not employed		540	45		431	43.6		152	45.2		117	41.5
Number of health events	.96 (1.24)			.96 (1.25)			.79 (1.07)			.75 (1.04)		
0		619	51.6		516	52.2		182	54.2		157	55.7
1		263	21.9		206	20.8		83	24.7		68	24.1
2		141	11.8		119	12		42	12.5		35	12.4
3		100	8.3		86	8.7		17	5.1		14	5
4		76	6.3		62	6.3		12	3.6		8	2.8

Note: [a] Reflects the number and percentage of participants answering “yes” to this question.

Table 2

Means, standard deviations and correlations between social variables and clinical sleep disturbance

		Correlations								
	M(SD)	1	2	3	4	5	6	7	8	9
Perceived support										
1. Total	3.49 (.42)	1								
2. Partner	3.64 (.51)	**.66****	1							
3. Family	3.52 (.59)	**.78****	**.31****	1						
4. Friends	3.32 (.61)	**.75****	**.21****	**.37****	1					
Perceived strain										
5. Total	1.97 (.42)	**-.41****	**-.43****	**-.33****	**-.17****	1				
6. Partner	2.13 (.61)	**-.46****	**-.65****	**-.25****	**-.15****	**.73****	1			
7. Family	2.00 (.58)	**-.27****	**-.15****	**-.34****	**-.10****	**.78****	**.29****	1		
8. Friends	1.77 (.48)	**-.17****	**-.11****	**-.14****	**-.12****	**.74****	**.28****	**.48****	1	
9. Clinical sleep disturbance	5.45(3.14)	**-.11****	**-.08***	**-.10****	-.06	**.14****	**.08****	**.16****	**.06***	1

Note: † *p*<.10.
* *p*<.05.
** *p*<.01.

Table 3

Means, standard deviations and correlations between social variables and daily diary and actigraphy sleep indicators

		Correlations													
	M(SD)	1	2	3	4	5	6	7	8	9	10	11	12	13	14
Perceived support															
1. Total	3.48 (.43)	1													
2. Partner	3.65 (.49)	**.66****	1												
3. Family	3.48 (.63)	**.78****	**.31****	1											
4. Friends	3.31 (.63)	**.76****	**.26****	**.35****	1										
Perceived strain															
5. Total	2.0 (.39)	**-.42****	**-.37****	**-.36****	**-.21****	1									
6. Partner	2.13 (.58)	**-.56****	**-.58****	**-.26****	**-.23****	**.67****	1								
7. Family	2.06 (.56)	**-.27****	-.10†	**-.37****	-.09	**.78****	**.21****	1							
8. Friends	1.82 (.48)	**-.15****	-.07	**-.13***	-.12†	**.73****	**.18****	**.48****	1						
Daily diary measured sleep outcomes															

9. Sleep disturbance	2.36 (.70)	-.24**	-.17**	-.19**	-.17**	.17**	.16**	.11†	.11†	1					
10. Light sleep	2.22 (.67)	-.21**	-.14*	-.17**	-.16**	.20**	.10†	.18**	.16**	.84**	1				
11. Less-rest	2.37 (.68)	-.25**	-.23**	-.19**	-.13*	.21**	.24**	.13*	.08	.85**	.67**	1			
Actigraphy measured sleep outcomes															
12. Sleep efficiency	83.45 (7.56)	.005	.03	.03	-.05	-.06	-.13*	.01	-.01	-.04	-.07	-.01	1		
13. Sleep onset latency	22.65 (19.51)	-.001	.004	-.05	.05	.03	.03	.03	.01	-.01	.01	-.01	-.73**	1	
14. Sleep time	386.25 (62.38)	0.00	.02	0.00	-.02	.01	-.11†	.07	.09	.01	-.01	.02	.59**	-.35**	1

Note: † *p*<.10.
* *p*<.05.
** *p*<.01.

Table 7

Adjusted estimates predicting daily sleep disturbance from different sources of perceived social support and total social strain

	Daily sleep disturbance			
	b(SE)	*p*	95% CI	
			LL	*UL*
Support from partner	-.12 (.09)	.21	-.37	.09
Support from family	-.10 (.07)	.18	-.27	.06
Support from friends	-.09 (.07)	.21	-.23	.06
Total perceived strain	.15 (.12)	.19	-.11	.38
R^2	.10			
Adj. R^2	.07			
F	2.8	.001		

Note: Adjusted for major health events, age, employment status, number of caffeinated drinks, number of alcoholic drinks, minutes of moderate or vigorous exercise, and length of nap time (minutes)

Table 8
Adjusted estimates predicting clinical sleep disturbance and subjective daily sleep outcomes from different sources of perceived social support and strain

	PSQI				Light sleep				Less rest			
	b(SE)	*p*	95% CI		*b(SE)*	*p*	95% CI		*b(SE)*	*p*	95% CI	
			LL	*UL*			*LL*	*UL*			*LL*	*UL*
Support from partner	-.17 (.26)	.53	-.69	.33	-.12 (.10)	.26	-.41	.14	-.14 (.10)	.16	-.45	.12
Support from family	-.18 (.19)	.35	-.53	.17	-.07 (.08)	.32	-.24	.09	-.08 (.07)	.30	-.25	.08
Support from friends	-.12 (.17)	.50	-.46	.24	-.10 (.07)	.15	-.25	.05	-.05 (.07)	.45	-.20	.11
Strain from partner	.11 (.22)	.61	-.34	.57	-.03 (.09)	.72	-.23	.14	.16 (.09)	.056	-.03	.34
Strain from family	.71 (.21)	.001	.31	1.11	.13 (.09)	.13	-.05	.33	.03 (.09)	.72	-.13	.21
Strain from friends	-.13 (.24)	.59	-.58	.35	.13 (.09)	.18	-.07	.31	.02 (.09)	.83	-.17	.20
R^2	.04				.07				.14			
Adj. R^2	.03				.03				.10			
F	4.26	<.001			1.70	.06			3.42	<.001		

Note: Adjusted for major health events, age, employment status, number of caffeinated drinks, number of alcoholic drinks, minutes of moderate or vigorous exercise, and length of nap time (minutes)
